# Detection of Methicillin-Resistant *Staphylococcus aureus* in Clinical and Subclinical Mastitis in Ruminants and Studying the Effect of Novel Green Synthetized Nanoparticles as One of the Alternative Treatments

**DOI:** 10.1155/2022/6309984

**Published:** 2022-11-22

**Authors:** Abeer Mostafa Abdalhamed, Gamil Sayed Gamil Zeedan, Amany Ahmed Arafa, Eman Shafeek Ibrahim, Doaa Sedky, Amani Abdel nabey Hafez

**Affiliations:** ^1^Department of Parasitology and Animal Diseases, National Research Centre, Dokki, Egypt; ^2^Department of Microbiology and Immunology, National Research Centre, Dokki, Egypt; ^3^Department of Animal Health, Division of Animal and Poultry Production, Desert Research Center, Matariya, Cairo, P. O. Box 11562, Egypt

## Abstract

Mastitis is an important disease in dairy animals worldwide. Methicillin-resistant *Staphylococcus aureus* (MRSA) is one of the most common causes of clinical and subclinical intramammary infections. In the current study, we isolated bacteria from 150 mastitic milk samples. Multiplex PCR was used to detect the methicillin resistance genes in *S. aureus* to detect the occurrence of MRSA isolates. Green synthesized titanium dioxide nanoparticles (TiO2 NPs) using aqueous leaf extracts of *Artemisia herb Alba* (*A. herb Alba* TiO2 NPs). The antibacterial efficacy of these nanoparticles was evaluated (in vitro and in vivo) against collected MRSA isolates using the disc diffusion method and SPF rats. Out of 150 mastitic milk samples, the frequency of *S. aureus* was 38 (25.3%), that of *E. coli* was 45 (30%), that of *Klebsiella* spp. Was 7 (4.7%), and that of *Streptococcus* spp. Was 11 (7.3%). Among 38 positive isolates of *S. aureus*, MRSA was 16 (42.1%) by antimicrobial sensitivity testing (AST) and 14 (38.8%) by multiplex PCR. The MRSA isolates were shown to have 100% resistance to penicillin and methicillin, 87.5% resistance to gentamicin, 50% resistance to cefoxitin and amoxicillin, and 75% resistance to ampicillin and ampicillin/sublactam with low resistance against erythromycin, ciprofloxacin, tetracycline, and levofloxacin by AST, respectively. *A. herb Alba* TiO2 NP formation was observed by changing the colour from white to dark green. The UV spectrum revealed absorbance peaks at 240–250 nm, and their sizes ranged from 42–66 nm and 11 to 45 nm by scanning electron microscopy (SEM) and transmission electron microscopy (TEM). ‏*A. herb Alba* TiO2 NP suspensions were evaluated against MRSA, with the highest zone of inhibition (43 ± 0.45 mm) at a concentration of 40 *μ*g/ml. Hematological parameters and histological examination after oral administration of 20 mg/kg of *A. herb Alba* TiO2 NPs indicated that *A. herb Alba* TiO2 NPs can be used as a new antimicrobial against resistant bacteria (MRSA) with consideration of the dose and methods of synthesis of plant-based compounds.

## 1. Introduction

Mastitis is a global challenge for dairy animals. It is associated with economic losses due to the reduction in milk production and quality because it affects animal health [1, 2].

The main causes of mastitis are bacterial infections. Among these bacteria are *Staphylococcus aureus* (*S. aureus*), *Klebsiella pneumoniae*, *Streptococcus* spp., *Escherichia coli* (*E*. *coli*), and other less common pathogens like *Pseudomonas aeruginosa*, *Mycoplasma* species, and *Mycobacterium* species [3, 4].

*Staphylococcus aureus* is one of the most common bacteria causing mastitis in dairy animals. It causes subclinical and clinical infection of the mammary glands, which spreads during the milking process and leads to chronic infections, especially with strains showing multidrug resistance. In addition, more than 10% of food-borne infections associated with dairy products are caused by enterotoxigenic *S. aureus* [5].

Multidrug-resistant (MDR) staphylococci are a growing problem, due to the rise of drug-resistant virulent strains of *S*. *aureus*, particularly methicillin-resistant *S*. *aureus* (MRSA), which is a serious problem in the treatment and control [6]. MRSA strains usually exhibit resistance to commonly used antibiotics, including macrolides, aminoglycosides, chloramphenicol, fluoroquinolones, and tetracyclines, due to the widespread use of antibiotics in veterinary field, which leads to the spread of antibiotic-resistant bacteria to humans [7]. The major emerging type of MRSA has been studied in livestock animals (LAs), which is known as livestock-associated MRSA (LA-MRSA). Different LA-MRSA strains have been found in different foods, including milk, cheese, meat products, and raw meat of chicken [8, 9].

MRSA infection management includes rapid and successful detection methods that begin with traditional cultural protocols and are confirmed by polymerase chain reaction (PCR)-based assays. However, these methods also have some complications, and it is necessary to run multiplex PCR to obtain reliable results [10] and update preventive protocols with great interest in the search for new antimicrobial substances such as plant-based synthesis of nanoparticles (NPs). The ideal solution to treat bacteria is the use of antibiotics. However, in recent years, there has been an increase in the resistance of different bacterial strains to these antibiotics. Therefore, there is currently a great interest in the search for new antimicrobial substances [11].

The interest in new antimicrobial substances has been focused on metal oxide NPs. Specifically, titanium dioxide (TiO2), which is considered an attractive antimicrobial compound due to its photocatalytic nature, is nontoxic, inexpensive, and stable NP [12].

The United States Food and Drug Administration (FDA) has demonstrated that titanium dioxide nanoparticles (TiO2 NPs) are nontoxic, and they have been used in different products such as food-grade TiO2 NPs, which are present in a wide range of food products like gum, candy, donuts, marshmallows, cookies, and toothpaste. Shaving creams, deodorants, conditioners, shampoos, and sunscreens are some of the personal care products containing TiO2 NPs [13].

Plant extracts have been considered as one of the best methods for metal oxide nanoparticle synthesis because they are fast reducing agents and produce stable NPs [14, 15].

Recent studies have reported the use of various plant species such as *Sesbania grandiflora* L. leaf extract, *Moringa oleifera*, *Aloe barbadensis*, *Calotropis gigantea*, *Ageratina altissima* L., *Curcuma longa*, and *Vitex negundo* in the synthesis of TiO2 NPs [16, 17]. In recent years, the fear of pan drug resistance (resistance to all antibiotics) has increased, so we are trying to find a new antimicrobial therapy for clinical mastitis (CM) and subclinical mastitis (SCM) caused by MDR *S. aureus* using green synthesis of metal oxide nanoparticles [18, 19]. Based on natural plant extracts, TiO2 NPs have broad-spectrum activity against Gram-negative and Gram-positive bacteria and fungi [20].

In this study, the isolation of bacteria in mastitic milk samples from dairy cows, buffaloes, sheep, and goats was determined, and resistance genes in positive isolates of *S. aureus* causing mastitis were detected using multiplex PCR to evaluate their resistant state (MRSA). We evaluated the antibacterial efficacy of these nanoparticles against isolated MRSA in vitro and in vivo, in order to develop new strategies for reducing the spread of these resistant pathogens using new green synthesized NPs. This is the first work to describe the preparation of *A. herb Alba* TiO2 NPs and investigate their antimicrobial effect against MRSA.

## 2. Materials and Methods

### 2.1. Ethics Statement

The experimental protocols and handling of experimental animals in this study were approved by Ethics Review Committee of National Research Centre, Egypt, under approval number: 19-149.

### 2.2. Collection of Samples and Study Localities

A total of 150 milk samples were collected from animals suffering from clinical and subclinical mastitis (cows: 55; buffaloes: 32) from Monofia, Giza, Beni Suef, Siwa, Marsa Matruh, and Alexandria in Egypt, from December 2019 to April 2020, as shown in Table 1. The milk samples were used for the isolation and identification of different bacteria causing mastitis. Subclinical mastitis cases were detected using the California mastitis test (CMT) by mixing 3 mL milk samples from each quarter and 3 mL CMT reagent using a partitioned plastic paddle, as previously described [21]; then, the selection of animals with clinical mastitis (CM) was based on swelling of the mammary glands and abnormalities in the milk (pus and blood streaks), positive CMT, and decreased milk yield [22]. The udders of animals were cleaned with water and dried using cotton with 75% ethanol before samples were collected.

### 2.3. Isolation and Phenotypic Identification of Bacteria from Mastitic Milk Samples

The collected milk sample was kept in a sterile tube and transported to the laboratory within 4 h. Milk samples were cultured on nutrient agar, blood agar, mannitol salt agar, and MacConkey's agar (Oxoid, Hampshire, UK), and then the cultured plates were incubated for 24–48 h at 37°C. The pure colonies were first identified according to their Gram staining, morphological characteristics, hemolysis, and biochemical characteristics as described in [23]. Positive isolates of *S. aureus* were examined by multiplex PCR to determine their MRSA status [24]. Isolates of *S. aureus* were kept at −20°C in tryptic soy broth (TSB; Becton Dickinson, Wokingham, UK), containing 20% volume of glycerol.

### 2.4. Antimicrobial Susceptibility Testing (AST) for Isolated *S. aureus* and MRSA Strain

Positive *S. aureus* isolates and identified MRSA strains were tested for their susceptibility to eleven antimicrobial agents using the standard disc diffusion method on Mueller–Hinton agar (Merck, Darmstadt, Germany), following 0.5 McFarland standards (1.5 × 10^8^). The test was performed and interpreted in accordance with the guidelines of the Clinical and Laboratory Standards Institute (CLSI) [25]. Eleven antimicrobial agents were included: penicillin G (PEN), Amoxicillin (Ax), ampicillin (AMP), gentamicin (GEN), ampicillin/sublactam (SAM), cefoxitin (Fox), methicillin (MET), erythromycin (ERY), ciprofloxacin (CIP), tetracycline (TET), and levofloxacin (LEV). The zone of inhibition was recorded in millimetres.

### 2.5. Molecular Identification

#### 2.5.1. DNA Extraction from Isolated *S. aureus*

All phenotypically isolated *S. aureus* strains were used to extract DNA by boiling methods. In brief, a pure colony was collected from freshly grown culture into an Eppendorf tube containing molecular-grade water (100 ul), followed by mixing gently. Then, the mixture was boiled for 10 minutes, cooled for 10 minutes, and centrifuged for 10 minutes at 2400 rpm. Finally, the supernatant was collected as the source for the genomic DNA; it was stored at −20°C until used for multiplex PCR to determine the MRSA status of *S. aureus* [26].

#### 2.5.2. Detection of MRSA among Isolates of *S. aureus* from Mastitic Milk by Multiplex PCR

Isolates showing resistance to some antimicrobial agents were investigated by multiplex PCR for amplification of *tetM* and *tetK* (tetracycline resistance), *mecA* (methicillin resistance), *ermB* (erythromycin resistance), and *blaZ* (penicillin resistance) genes using primers as shown in Table 2. Multiplex PCR was performed in a gradient thermal cycler (Eppendorf, Hamburg, Germany) [27]. Each 25 *μ*l reaction mixture contained 2 *μ*l of genomic DNA, 12.5 *μ*l of PCR master mix (Promega Corporation, Madison, WI, USA), and 0.5 *μ*l of 100 pmol of the forward and reverse primers and was adjusted with 5.5 *μ*l of nuclease-free water to complete 25 *μ*l final volumes. Denaturation at 94°C for 5 minutes was followed by 30 cycles of 94°C for 1 minute, 55°C for 30 seconds, and 72°C for 1 minute, with a final step of elongation at 72°C for 5 minutes. The PCR products were examined by 1% agarose gel electrophoresis (Germany), with ethidium bromide staining, and visualized by UV transilluminator according to the methods.

### 2.6. Green Synthesis of *Artemisia herb Alba*-Based Titanium Dioxide (TiO2)

#### 2.6.1. Preparation of *A. herb Alba* Aqueous Leaf Extract

Clean leaves of *A. herb Alba* were washed using distilled water, dried in air, and ground into a fine powder via a blender. Aqueous leaf extract of *A. herb Alba* was heated for 20 min at 80°C under continuous stirring and then at 46°C for 24 h. Subsequently, it was filtrated and kept for use in a refrigerator.

#### 2.6.2. Preparation of Green Synthesis of *A. herb Alba* TiO2 NPs

The green synthesis of TiO2 NPs was prepared according to the methods described in [28]. In brief, for the synthesis of *A. herb Alba* TiO2 NPs, 25 mL of aqueous extract of *A. herb Alba* was mixed with 225 mL of a 5 mM (TiO2) solution and incubated for 24 h under light conditions at room temperature. A greenish colour was developed, which confirms the generation of TiO2 NPs. The prepared TiO2 NPs were separated by centrifugation (15,000×*g*, 20 min), washed thrice with distilled water, and dried overnight at 120°C to obtain a porous fine powder that was characterized to confirm the success of NP preparation.

#### 2.6.3. Characterization of *Artemisia herb Alba*-Based TiO2 NPs

*(1) UV Visible Spectrophotometer*. The formation and bio-reduction of *A. herb Alba* TiO2 NPs were monitored by a UV-visible spectrophotometer (UV-vis spectra) of synthesized NPs [29] at the National Research Centre (NRC), Egypt.

*(2) Electron Microscopy*. The size of TiO2 NPs particles was evaluated using transmission electron microscopy (TEM). The grid was prepared by placing an aqueous suspension of *A. herb Alba* TiO2 NPs on a TEM grid and drying in air overnight before imaging. Separate images were taken at magnifications of 20,000xs to 100,000xs at NRC using a TEM unit; SigmaScan Pro software was used to measure the average size of *A. herb Alba* TiO2 NPs [30]. In scanning electron microscopy (SEM) analysis, a fine powder of *A. herb Alba* TiO2 NPs was used. The images were recorded using SEM at NRC, Egypt, to observe the shape and size of the green synthesized NPs [31].

#### 2.6.4. Antibacterial Activity of *A. herb Alba* TiO2 NPs

The antibacterial activity of synthesized *A. herb Alba* TiO2 NPs was evaluated against isolated MRSA by using the disc and agar diffusion method. A standard inoculum of bacterial isolates at a concentration of 1.5 × 10^8^ CFU/mL was formed and compared with the standard solution of 0.5 McFarland. They were cultivated on Mueller–Hinton medium and impregnated with sterile discs [32–34]. The discs were filled with *A. herb Alba* TiO2 NPs with various concentrations (5, 10, 20, 30, and 40 *μ*g/ml), and an empty disc with sterile distilled water was used as a negative control. Inhibition zones were measured after incubation with the inoculated plate at 37°C for 24 h.

### 2.7. In Vivo Experiments to Study the Effect of *Artemisia herb Alba*-Based Titanium Dioxide Nanoparticles

#### 2.7.1. Experimental Animals

Twenty adult male and female healthy SPF Webster rats weighing between 150 and 190 g were used in this research. They were obtained from the animal house of NRC, Cairo, Egypt, and randomly divided into four groups for the experiment. All rats were transported to randomly chosen polypropylene cages containing sawdust sheets with net wire tops in a filtered-air room. They were kept under daily observation at a normal room temperature (20–25°C) and a relative humidity of 60–70% and received food and water [35]. SPF Webster rats were randomly divided into four groups, with five animals in each group. Group 1 (G1): healthy control rats were treated with saline (negative control); group 2 (G2): rats (S/C) were injected twice in the back with 0.2 ml of (3.0 × 107 CFU/l) cell suspension of MRSA in saline/rat for two days (positive control); group 3 (G3): MRSA infected group rats were orally administered ciprofloxacin (20 mg/kg BW); group 4 (G4): on the 21st day, all rats were weighed and blood was collected after being anaesthetized with chloroform by the ocular sinus puncture method. Then, the blood samples were stored at room temperature for hematological analysis.

#### 2.7.2. Hematological Analysis

Whole blood samples were immediately collected in an ethylene diamine tetra acetic acid (EDTA)-coated vacutainer for hematological analysis. Hematological parameters including hemoglobin (Hb), erythrocytes (RBC), white blood cell count (WBC), number of neutrophils (NEUT), number of lymphocytes (LYM), number of (monocytes, eosinophils, and basophils), platelet count (PLT), hematocrit (HCT%), mean corpuscular volume (MCV), mean corpuscular hemoglobin (MCH), mean corpuscular hemoglobin (MCH), mean corpuscular hemoglobin (MCH), mean corpuscular hemoglobin (MCHC), red cell distribution width (RDW), and mean platelet volume (MPV) were analyzed using an auto-hematology analyzer according to [36].

#### 2.7.3. Histological Examination

Tissue samples of the lungs, liver, and kidney of each rat were collected and immersed directly in 10% neutral buffered formalin for histopathological investigations. After 72 hours, the samples were processed, embedded in paraffin wax, and sectioned (5 ml) for hematoxylin and eosin (H&E) staining according to [37]. Histological slides were examined and photographed by a light microscope camera.

### 2.8. Statistical Analysis

The data were analyzed using Student's *t*-test and the data were expressed as mean ± division (SD) The value of *p* < 0.05 was considered as a significant value against the control.

## 3. Results

### 3.1. Frequency of Bacteria in Mastitic Milk Samples

Out of 150 mastitic milk samples, 38 (25.3%), 45 (30%), 7 (4.7%), and 11 (7.3%) *S. aureus*, *E. coli*, *Klebsiella* spp., and *Streptococcus* spp. isolates were recovered, respectively. Among the 38 *S*. *aureus* isolates, 16 (42.1%) were identified as MRSA by antimicrobial sensitivity testing (AST) and 14 (38.8%) by multiplex PCR from dairy animals in different governorates of Egypt, as shown in Table 3. *Staphylococcus aureus* was isolated from mastitic milk samples of dairy animals based on staining, golden yellowish colonies on MSA plates. The isolates showed complete (*β*) hemolysis on blood agar and coagulase tests, in addition to biochemical tests, which were all confirmed as positive isolates of *S. aureus*.

The patterns of antimicrobial sensitivity in Table 4 exhibited that out of 38 *S. aureus* isolates, 16 isolates (MRSA) were resistant to common available antibiotics. All 16 isolates were 100% resistant to penicillin G and methicillin, 87.5% resistant to gentamicin, 75% resistant to amoxicillin, ampicillin, and ampicillin/sublactam, 50% resistant to cefoxitin, 37.5% resistant to erythromycin, 31% resistant to ciprofloxacin, and 25% resistant to tetracycline and levofloxacin.

### 3.2. Identification of MRSA in Mastitic Milk

Multiplex PCR was used to detect methicillin resistance genes (*mec*A 331pb and *mec*A 314pb), the -lactamase gene *bla*Z (173 bp), erythromycin ribosome methylase genes (*erm*B142 bp), and tetracycline resistance genes *tet*M (406 bp) and tet K (158 bp). Among the 38 positive *S. aureus samples*, the positive detection rates for MRSA were 16 (42.1%) by AST and 14 (38.8%) by multiplex PCR as shown in Table 5. Multiplex PCR for confirmation of 16 phenotypically isolated MRSA isolates was performed, and it was found that 6 (37.5%) carried the *mecA* gene (331 pb), 5 (31.25%) carried the *mecA* gene (314 pb), 6 (37.5%) carried the *blaZ* gene (173 bp), 6 (37.5%) carried the *ermB* gene (142 bp), 6 (37.5%) carried the *tetM*gene (406 bp), and 1 (6.25) carried the *tet K* (158 bp).

Table 5 revealed that between 38 positive *S. aureus samples*, the positive detection rates for MRSA were 16 (42.1%) by AST and 14 (38.8%) by multiplex PCR. While, Multiplex PCR for confirmation of MRSA was 14 (36.8%)

### 3.3. Characterization of Green Synthesis of *A. herb Alba* TiO2 NPs

#### 3.3.1. Optical Observation of *A. herb Alba* TiO2 NPs

**Figure 1:**
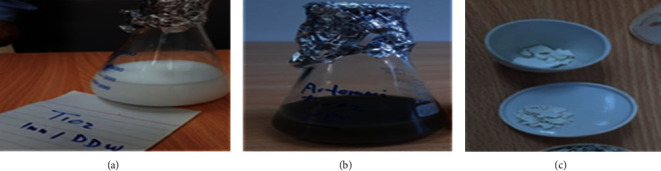
Formation of TiO2 NPs using *A. herb Alba* aqueous extract. (a) TiO2 solution. (b) *A. herb Alba* TiO2 NP powder. (c) TiO2 NP powder.

The colour of the colloidal solution changed from white to dark green, which indicated the excellent formation of titanium dioxide nanoparticles as shown in Figures 1(a) and 1(b); after centrifugation, washing, and drying overnight at 120°C, the obtained fine powder was used to characterize (Figure 1(c)).

#### 3.3.2. UV-Vis Spectra Analysis

**Figure 2:**
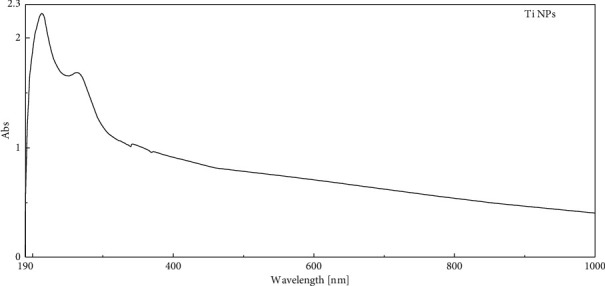
The UV-vis shows the spectrum of TiO2 NPs synthesized using *A. herb Alba* aqueous leaf extract.

The formation of TiO2 NPs in this study was analyzed by UV-visible spectrophotometer (Figure 2). The surface plasmon resonance (SPR) of the synthesized NPs appears as peaks due to the characteristic SPR of TiO2 NPs. The absorption spectra of *A. herb Alba* TiO2 NPs formed in the solution had absorbance peaks at around 240–250 nm for aqueous plant extract exposed to TiO2.

### 3.4. Electron Microscopy of *A. herb Alba* TiO2 NPs

**Figure 3:**
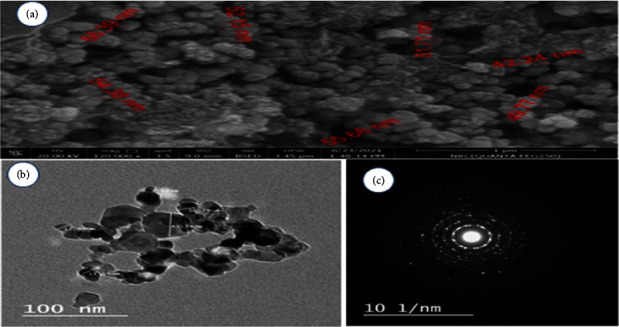
(a) Micrographs of *A. herb Alba* TiO2 NPs using SEM showed crystalline rods and relatively spherical shapes of the green synthesized NPs, with an average size of 42–66 nm. (b) TEM images of TiO2 NPs synthesized with *A. herb Alba* TiO2 NP aqueous leaf extract. (c) Selected area electron diffraction (SAED) pattern transmission electron microscopy is the most frequently reported technique to analyze the structure, size, and shape of the synthesized NPs.

The shape and size of nanoparticles were investigated. The SEM and TEM examinations were carried out to understand the morphology and size distribution of titanium dioxide nanoparticles. The SEM micrograph of the TiO2 NPs synthesized showed that there was a dense agglomeration of particles and they were irregular in shape. TEM reflection and size allocation of TiO2 NPs, which were a mixture of diverse sizes and shapes, the composition of particles as shown in Figure 3.

### 3.5. Antibacterial Effect of *A. herb Alba* TiO2 NPs

**Figure 4:**
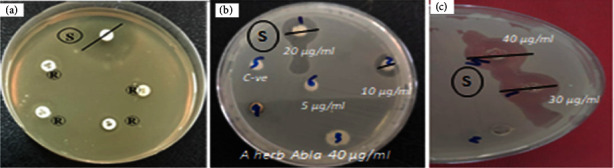
(a) An antibiotic sensitivity test for isolated *S. aureus* shows resistance (R) and sensitivity (S) of MRSA to different antibiotics. (b, c) Inhibition zone diameter (IZD) against isolated MRSA using leaf extract of *A. herb Alba* and *A. herb Alba* TiO2 NPs.

The antibacterial activity of various concentrations of synthesized *A. herb Alba* TiO2 NPs was evaluated against MRSA using disc diffusion assay. The mean inhibition zone (in millimetres) around each disc was estimated (Figures 4(a)–4(c)).

The ability of *A. herb Alba* TiO2 NPs, leaf extract of *A. herb Alba*, and antibiotics to inhibit bacterial growth of MRSA is listed in Table 6. The highest zone of inhibition (43 ± 0.45 mm) was observed at the highest concentration of *A. herb Alba* TiO2 NPs (40 *μ*g/ml).

### 3.6. Results of Oral Administration of *A. herb Alba* TiO2 NPs on Experimental Rats

No death or toxic symptoms were observed due to administration of *A. herb Alba* TiO2 NPs during the experiment period; the use of *A. herb Alba* TiO2 NPs for 20 days did not cause any harmful effects on body weight or growth.

#### 3.6.1. Hematological Analysis

Hematological parameters were analyzed to differentiate between rats that were treated with *A. herb Alba* TiO2 NP antibiotics after experimentally infected with MRSA and those that were not treated, as shown in Table 7. Rats infected with MRSA without treatment (G2) have shown a decrease in Hb when compared to control group rats (G1) and treated rats (G3 and G4). There was an increase in WBC in the *A. herb Alba* TiO2 NP treated group (G4). However, the total number of lymphocytes was higher in the infected group (G2) than treated group with antibiotics (G3), while the total number of granulocytes after infection had no significant changes in their levels when compared to the control group (*p* > 0.05). The number of PLTs decreased in the infected group (G2), and it is relatively normal in antibiotics and *A. herb Alba* TiO2 NP treated groups (G3 and G4).

Table 7 showed significant change (*p* > 0.05) in (monocytes, eosinophils, and basophils) between infected group (G2) and control negative group (G1). The results showed that the number of NEUT in (G2) was 0.52 (10^3^/cm), while in the control group (G1), it was 0.87 (10^3^/cm) which recorded significant decrease. The number of LYM showed a significant increase in (G2) after infection reaching 13.00 (10^3^/cm) when compared to the control negative (G1) which reached 7.3 (10^3^/cm).

#### 3.6.2. Examination of Rats' Tissue Section of the Lung, Liver, and Kidney by Light Microscopy

**Figure 5:**
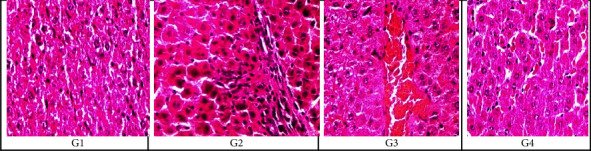
H&E stain showing the histological structure of liver tissue section at 20x magnification in the form of (G1) negative control group, (G2) rats infected with MRSA without treatment, (G3) rats treated with antibiotics and (G4) rats treated with *A. herb Alba* TiO2 NPs.

**Figure 6:**
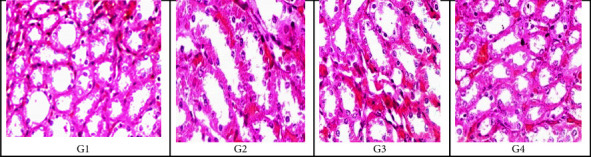
H&E stain showing the histological structure of kidney tissue section at 20x magnification in the form of (G1) negative control group, (G2) rats infected with MRSA without treatment, (G3) rats treated with antibiotics, and (G4) rats treated with *A. herb Alba* TiO2 NPs.

Specimens of lung sections from the control negative group (G1) using H&E staining magnification (x20) showed normal histological features of the lung. Specimens of the lung section of an infected group (G2) with MRSA showed thickening of the inter-alveolar septum, catarrhal bronchitis, infiltration with inflammatory cells, lymphocytic aggregates, hemorrhage in the bronchi, and interstitial pneumonia. Specimens of lung sections of (G3) rats showed that the pulmonary blood vessels were dilated, thickened, congested, and about to rupture. Specimens of lung sections of rats treated with *A. herb Alba* TiO2 NPs (G4) showed infiltration with inflammatory cells, alveolar emphysema with characteristic giant alveoli and bronchitis, thick inter-alveolar septa, and narrowing of the alveolar sac. The histological pattern of the lung tissue treated with *A. herb Alba* TiO2 NPs was the best, and the pattern of the lung tissue histologically was similar to the control negative group as shown in Figure 5. Specimens of liver sections from control negative (G1) rats' livers showed normal tissues of hepatic cells using H&E staining. The infected group with MRSA (G2) rat liver showed focal areas of congestive necrosis infiltrated by leucocytes and slight vacuolar degeneration of some hepatic cells. In rats treated with antibiotics (G3), liver sections showed centro lobular congestion surrounded by slight degeneration of hepatocytes. In rats treated with *A. herb Alba* TiO2 NPs (G4), liver sections showed blood sinusoids with slight degeneration of hepatocytes as shown in Figure 5. The kidney sections of the negative control group (G1) showed a normal picture of glomeruli, and rat kidneys infected with MRSA (G2) showed interstitial hemorrhage, necrosis in tubules, and interstitial infiltration of inflammatory cells; rats treated with antibiotics (G3) showed hemorrhage and degenerated tubules and glomeruli, and rats treated with *A. herb Alba* TiO2 NPs (G4) showed apparently normal renal tubules and glomeruli with slight hemorrhage and congestion as shown in Figure 6.

## 4. Discussion

**Figure 7:**
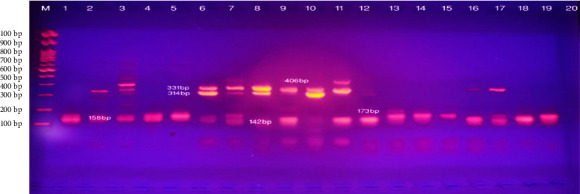
Shows a multiplex PCR assay to identify the *S. aureus* resistance genes *ermB* (142 bp), *tet K* (158 bp), *blaZ* (173 bp), *mecA* (314 bp), *mecA* (331 bp), and *tet M* (406 bp).Marker 100 Pb.

Livestock is the major reservoir of MRSA infection, with specific genotypic and phenotypic traits of 3% *S. aureus* infection [38–40]. The prevalence of *S. aureus* associated with intramammary infections in CM and SCM mastitis in dairy animals was 25.3% as shown in Tables 3 and 4, while other studies in Egypt (35.9%) [41, 42], and disagree with the lower prevalence (6.5%) reported by [40, 43, 44]. In contrast, the percent of S. aureus infections associated with CM and SCM mastitis compared to other previous studies may be due to biofilm formation, beside differences in immunological status of the animals, isolation protocols, geographical distribution, and hygienic protocols of the study areas. The frequency of MRSA infections increased steadily after the introduction of *β*-lactam antimicrobials, as shown in Table 4 [45]. The prevalence of MRSA was determined by S. aureus isolates from cows, buffaloes, sheep, and goats, suffering from CM and SCM mastitis more than percentage previously recorded in Egypt, may be due to the fact that this study used molecular techniques for detection of MRSA as shown in Table 5 [46]. The increase in the prevalence of MRSA may be due to the widespread usage of antimicrobials in recent years [47]. Confirmation of resistants' genes in the collected MRSA isolates, including *mecA* (331pb/314pb)*, blaZ*, *ermB*, *tetM*, and *tetK* by multiplex PCR as shown in Figure 7.

On analysis of *S. aureus* sensitivity showed resistance to penicillin G and methicillin was 100% and this result is in agreement with [48–50]. *S. aureus* resistance to *β*-lactam antibiotics is due to *mecA* gene, which encodes a modified penicillin binding protein that confers resistance to methicillin and other penicillin derivatives carried on a mobile gene element called the “staphylococcal cassette-chromosome-mec” (SCC-mec) [51–54]. The 75% of MRSA isolates sensitive to tetracycline and levofloxacin by AST is in agreement with [55], resistant to penicillin up to 100%, and cefoxitin 42.7% [56–58]. Development of green synthesized *A. herb Alba* TiO2 NPs, the formation of TiO2 NPs in aqueous solution was confirmed by UV-visible absorption, TEM images of *A. herb Alba* TiO2 NPs showed crystallite and hexagonal particles, with an average size of 11 to 45 nm as shown in Figures 3(a)–3(c) as reported with [59, 60]. *A. herb Alba* TiO2 NPs suspensions against MRSA, found that these Nano formulations are effective inhibitory effect of various concentrations against MRSA as exhibited in Figures 4(a), 4(b)4(c) and 5 [61, 62]. Who recorded that biosynthesized *Trigonella foenum graecum* leaf TiO2 NPs appeared as spherical NPs with a size of 20-90 nm. The highest zone of inhibition (43 ± 0.45 mm) was observed at the highest concentration of *A. herb Alba TiO2* NPs (40 *μ*g/ml) [63, 64].

The green TiO2 NPs had antibacterial activity by forming superoxide radicals (O2), which produce reactive oxygen species (ROS) in bacterial cells, leading to breaking of the bacterial cell membrane [65]. Another reason for the antibacterial activity of TiO2 NPs is their ability to inhibit the biofilm in multidrug-resistant bacteria by inducing cell lysis and destructing the cell wall of these bacteria, leading to cell death and eventually inhibiting biofilm formation [66, 67].

Hematological parameters changes, *A. herb Alba* TiO2 NPs experimentally treatment of infected rats group retained hematological parameters to normal values as control negative group as they have the ability to control the infection in 20 days and the high oxidizing power of the free radicals, which can directly destroy bacteria cells and inhibit the activity of ATPase, which leads to the reduction of the essential ATP to reserve the bacterial cell life and significant inhibitory effect on bacteria lead to recovery infected group rates to normal values as in group (G4) treated with *A. herb Alba* TiO2 NPs leads to a significant improvement in Hb, RBC, HCT, MCV, and MCHC levels as a results with [68]. The group treated with *A. herb Alba* TiO2 NPs showed a significant increase in the neutrophil numbers of 0.72 (10^3^/cm) compared to control infected groups (G2) without treatment and a reduction in the number of lymphocytes of 7.40 (10^3^/cm) in (G4) as shown in Table 7 [69, 70]. Green synthesized TiO2 NPs have hydroxyl and superoxide anions that disturb the cell wall and reduce the growth of several microorganisms, such as *E. coli* and *S. aureus*.

**Figure 8:**
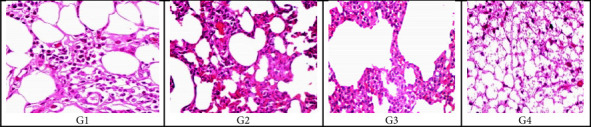
H&E stain showing the histological structure of the lung tissue section at 20x magnification in the form of (G1) negative control group, (G2) rats infected with MRSA without treatment, (G3) rats treated with antibiotics and (G4) rats treated with *A. herb Alba* TiO2 NPs.

Histopathological examination of infected groups with MRSA revealed an effect on lung tissue because it caused thickening of the inter-alveolar septum, infiltration with inflammatory cells, catarrhal bronchitis, lymphocytic aggregates, lymphocytic granuloma, hemorrhage in the bronchi, and interstitial pneumonia in the lung. Furthermore, in the liver, it causes congestive necrosis and vacuolar degeneration of some hepatic cells, as shown in Figures 5, 6, and 8, and interstitial hemorrhage and infiltration of inflammatory cells in the kidney. Using *A. herb Alba* TiO2 NPs for the treatment of MRSA decreased the severity of damage in tissues but did not return to normal. Therefore, more studies are necessary to determine its toxicity. In this study, laboratory rats that had been experimentally infected with MRSA strains and treated with *A. herb Alba* TiO2 NPs showed an improvement in the values of hematological parameters, in addition to the significant restoration of the rates of WBCs, lymphocytes, and granules after treatment [71, 72]. *A. herb Alba* TiO2 NPs had antimicrobial property for treatment of clinical and subclinical mastitis against multidrug-resistant bacteria especially MRSA.

## 5. Conclusion

The current study was conducted to develop possible alternative economically therapeutic agents from green synthesis of *A. herb Alba* TiO2 NPs for curing clinical and subclinical mastitis in ruminants in Egypt, for which conventional antimicrobial therapy may fail to provide a response. The highest rates of MRSA strains from positive *S. aureus* isolates were detected by AST and multiplex PCR assays. Phenotypically, resistance of positive isolates of *S. aureus* against 11 available antibiotics showed 100% resistance to penicillin and methicillin. The most predominant resistance genes associated with MRSA strains in mastitic milk were *mecA*, *bla*Z*, ermB*, *tetM*, and *tetK.* The green *A. herb Alba* TiO2 NPs at concentration of 40 *μ*g/mL (average size of 11 to 45 nm) on MRSA-induced mastitis *in vitro*. While, *in vivo* study evaluated the efficacy of *A. herb Alba* TiO2 NPs in different hematological parameter, and histopathological examination in different organs of rats in different groups. The study findings support that *A. herb Alba* TiO2 NP green synthesized can be used as a novel antimicrobial therapeutic agent in MRSA causing clinical and subclinical mastitis. However, further studies are still needed to safety and toxicity at a level of genome and cells must be conducted in order to better comprehend the nano interactions with organisms and environment.

## Figures and Tables

**Table 1:** Samples collected from different dairy animals.

Location or governorate	Cows		Buffaloes		Location or governorate	Sheep		Goats	
	SCM	CM	SCM	CM		SCM	CM	SCM	CM
Monofia	10	4	7	2	Siwa	10	3	9	2
Giza	13	3	8	2	Marsa Matruh	6	4	6	3
Beni Suef	14	2	10	3	Alexandria	12	4	10	3
Total	37	9	25	7	Total	28	11	25	8
	**46**		**32**			**39**		**33**	

Clinical mastitis = CM; subclinical mastitis = SCM.

**Table 2:** PCR primers used to detect antimicrobial resistance genes in MRSA isolates.

Isolated bacteria	Target genes	Primer sequence (5′-3′)	Size of amplicon (bp)	References
Methicillin-resistant *Staphylococcus aureus*	*tet(M)*	F—AGTGGAGCGATTACAGAA R—CATATGTCCTGGCGTGCTTA	406 bp	[6–11, 27]
	*tet(K)*	*F—*GTAGCGACAATAGGTAATAGT *R—*GTAGTGACAATAAACCTCCTA	158 bp	
	*mecA*	F—CCTAGTAAAGCTCCGGA R—CTAGTCCATTCGGTCCA	331 bp	
	*mecA*	F—CCTAGTAAAGCTCCGGAA CCTAGTAAAGCTCCGGAA R—CTAGTCCATTCGGTCCA	314 bp	
	*ermB*	F—CTATCTGATTGTTGAAGAAGGATT R—GTTTACTCTTGGTTTAGGATGAAA	142 bp	
	*blaZ*	*F—*ACTTCAACACCTGCTGCTTTC *R—*TGACCACTTTTATCAGCAACC	173–174	

1F = forward; *R* = reverse.

**Table 3:** The percentage of bacteria isolated from mastitic milk samples of different animals.

Species	Mastitic milk samples	Characterization of bacterial species by gold standard culture methods (+ve)							
		*S. aureus*		*E. coli*		*Klebsiella* spp.		*Streptococcus* spp.	
		+ve	%	+ve	%	+ve	%	+ve	%
Cows	46	9	19.5	11	23.9	2	4.3	4	8.6
Buffaloes	32	7	21.8	13	40.6	2	6.2	2	6.2
Sheep	39	12	30.7	10	25.6	2	5.1.2	3	7.7
Goats	33	10	30.3	11	33.3	1	3.3	2	6.0
Total	150	38	25.3	45	30	7	4.66	11	7.33

**Table 4:** Frequency of antibiotic resistance of 16 isolates of MRSA.

Antimicrobial agents	PEN	Ax	AMP	GEN	SAM	Fox	MET	ERY	CIP	TET	LEV
No. (%) of MRSA isolates from mastitic milk (*n* = 16)	16 (100)	12 (75)	12 (75)	14 (87.5)	12 (75)	8 (50)	16 (100)	6 (37.5)	5 (31)	4 (25)	4 (25)

Penicillin (PEN), amoxicillin (Ax), ampicillin (AMP), gentamicin (GEN), ampicillin/sublactam (SAM), cefoxitin (Fox), methicillin (MET), erythromycin (ERY), ciprofloxacin (CIP), tetracycline (TET), and levofloxacin (LEV).

**Table 5:** Evaluation of *S. aureus* and MRSA causing mastitis in animals by multiplex PCR and AST.

Dairy animals	No. of +ve *S. aureus*	MRSA by AST (%)	MRSA by multiplex PCR (%)
Cows	9	4 (44.4)	3 (33.3)
Buffaloes	7	3 (42.8)	2 (28.5)
Sheep	12	5 (41.6)	5 (41.6)
Goats	10	4 (40)	4 (40)
Total	38	16 (42.1)	14 (36.8)

**Table 6:** Inhibition zone diameter of *A. herb Alba* TiO2 NPs against MRSA.

Items	MRSA/IZD (mm ± SD)				
Concentration	5 *μ*g/ml	10 *μ*g/ml	20 *μ*g/ml	30 *μ*g/ml	40 *μ*g/ml
*A. herb Alba* TiO2 NPs	6 ± 0.23	16 ± 0.45	26 ± 0.53	40 ± 0.65	43 ± 0.45
Leaf extract of *A. herb Alba*	12 ± 0.35				
Antibiotics	55 ± 0.65				

**Table 7:** Infected rats' whole blood picture following treatment with *A. herb Alba* TiO2 NPs and antibiotics.

Blood parameters	Group (G1) M. vale ± SD	Group (G2) M. vale ± SD	Group (G31) M. vale ± SD	Group (G4) M. vale ± SD
Hb (g/dL)	14.8 ± 0.034	12.7 ± 0.56	14.6 ± 0.287	14.2 ± 0.87
RBC (10^6^/UL)	8.5 ± 0.933	7.9 ± 0.234	8.1 ± 0.023	8.2 ± 0.023
WBC (10^3^/UL)	10.2 ± 0.533	12 ± 0.25	11.4 ± 0.02	13.30 ± 0.34
Neutrophils (10^3^/cm)	0.87 ± 0.45	0.52 ± 0.65	1.0 ± 0.02	0.72 ± 0.02
Lymphocytes (10^3^/cm)	7.3 ± 0.34	13.00 ± 0.5	11.0 ± 0.03	7.40 ± 0.023
Monocytes (10^3^/cm)	0.33 ± 0.03	0.36 ± 0.01	0.15 ± 0.04	0.10 ± 0.01
Eosinophils (10^3^/cm)	0.00 ± 0.00	0.03 ± 0.00	0.12 ± 0.2	0.01 ± 0.0
Basophils (10^3^/cm)	0.00 ± 0.0	0.00 ± 0.00	0.02 ± 0.01	0.01 ± 0.0
PLT (10^3^/UL)	554 ± 0.06	448 ± 0.93	590 ± 0.345	603 ± 1.56
HCT (%)	46.9 ± 0.34	44.8 ± 0.83	46.3 ± 0.98	46.4 ± 98.0
MCV (fL)	59 ± 0.56	57 ± 0.62	58.3 ± 0.54	58.2 ± 0.0876
MCH (pg)	20.0 ± 0.56	17 ± 0.54	20 ± 0.32	21 ± 0.98
MCHC (g/dL)	32 ± 0.567	29 ± 0.84	30 ± 0.98	31 ± 0.35
RDW-CV (%)	12.40 ± 0.345	10.6 ± 0.93	11.7 ± 0.56	11.8 ± 0.98
MPV (fL)	7 ± 0.123	6.5 ± 0.25	7.07 ± 0.23	6.30 ± 0.65

Mean values of different parameters ± stander division (SD) and all values significant at (*p* ≤ 0.05) Approx. highlysignificant at (*p* ≤ 0.001). (G1): negative control; (G2): rats were infected with MRSA without treatment (positive control); (G3):rats infected with MRSA and treated with ciprofloxacin; and (G4):rats infected with MRSA and treated *A. herb Alba* TiO2 NPs.

## Data Availability

All data generated or analyzed during this study are available from the corresponding author upon request.
